# Acute kidney injury risk prediction score for critically-ill surgical patients

**DOI:** 10.1186/s12871-020-01046-2

**Published:** 2020-06-03

**Authors:** Konlawij Trongtrakul, Jayanton Patumanond, Suneerat Kongsayreepong, Sunthiti Morakul, Tanyong Pipanmekaporn, Osaree Akaraborworn, Sujaree Poopipatpab

**Affiliations:** 1 Critical Care Division, Internal Medicine Department, Faculty of Medicine Varjia Hospital, Navamindradhiraj University, Bangkok, Thailand; 2 Clinical Epidemiology Department, Faculty of Medicine, Thammasat University, Pathum Thani, Thailand; 3 Center for Clinical Epidemiology and Clinical Statistics, Faculty of Medicine, Chiang Mai University, Chiang Mai, Thailand; 4 Anesthesiology Department, Faculty of Medicine Siriraj Hospital, Mahidol University, Bangkok, Thailand; 5 Anesthesiology Department, Faculty of Medicine Ramathibodi Hospital, Mahidol University, Bangkok, Thailand; 6 Anesthesiology Department, Faculty of Medicine, Chiang Mai University, Chiang Mai, Thailand; 7 Surgery Department, Faculty of Medicine, Prince of Songkla University, Songkhla, Thailand; 8 Anesthesiology Department, Faculty of Medicine Vajira Hospital, Navamindradhiraj University, Bangkok, Thailand

**Keywords:** Acute kidney injury, Risk prediction score, Critically-ill surgical patient, Intensive care unit

## Abstract

**Background:**

There has been a global increase in the incidence of acute kidney injury (AKI), including among critically-ill surgical patients. AKI prediction score provides an opportunity for early detection of patients who are at risk of AKI; however, most of the AKI prediction scores were derived from cardiothoracic surgery. Therefore, we aimed to develop an AKI prediction score for major non-cardiothoracic surgery patients who were admitted to the intensive care unit (ICU).

**Methods:**

The data of critically-ill patients from non-cardiothoracic operations in the Thai Surgical Intensive Care Unit (THAI-SICU) study were used to develop an AKI prediction score. Independent prognostic factors from regression analysis were included as predictors in the model. The outcome of interest was AKI within 7 days after the ICU admission. The AKI diagnosis was made according to the Kidney Disease Improving Global Outcomes (KDIGO)-2012 serum creatinine criteria. Diagnostic function of the model was determined by area under the Receiver Operating Curve (AuROC). Risk scores were categorized into four risk probability levels: low (0–2.5), moderate (3.0–8.5), high (9.0–11.5), and very high (12.0–16.5) risk. Risk of AKI was presented as likelihood ratios of positive (LH+).

**Results:**

A total of 3474 critically-ill surgical patients were included in the model; 333 (9.6%) developed AKI. Using multivariable logistic regression analysis, older age, high Sequential Organ Failure Assessment (SOFA) non-renal score, emergency surgery, large volume of perioperative blood loss, less urine output, and sepsis were identified as independent predictors for AKI. Then AKI prediction score was created from these predictors. The summation of the score was 16.5 and had a discriminative ability for predicting AKI at AuROC = 0.839 (95% CI 0.825–0.852). LH+ for AKI were: low risk = 0.117 (0.063–0.200); moderate risk = 0.927 (0.745–1.148); high risk = 5.190 (3.881–6.910); and very high risk = 9.892 (6.230–15.695), respectively.

**Conclusions:**

The function of AKI prediction score to predict AKI among critically ill patients who underwent non-cardiothoracic surgery was good. It can aid in early recognition of critically-ill surgical patients who are at risk from ICU admission. The scores could guide decision making for aggressive strategies to prevent AKI during the perioperative period or at ICU admission.

**Trial registration:**

TCTR20190408004, registered on April 4, 2019.

## Background

Acute kidney injury (AKI), a rapid deterioration of kidney function, is one of the most common complications affecting major surgical patients admitted to the intensive care unit (ICU) [1, 2]. Occurrences of AKI in critically-ill surgical patients are independently associated with increased length of ICU stay, morbidity, and mortality [1, 2].

Currently, several studies that attempted to identify AKI from an early stage using biomarkers have been reported [3]. However, the commercial biomarkers for detecting AKI remain unobtainable in many countries.

Another option for providing AKI prediction scores has been postulated for improving early diagnosis of AKI [4]. The AKI prediction scores have been developed from various population groups; for instance, cardiothoracic surgical patients [5, 6], general surgical patients [7, 8], or mixed medical and surgical critically-ill patients [9]. However, it is rarely reported from the perspective of non-cardiothoracic critically-ill surgical patients, whose illness severity is worse than general surgical patients, and surgical interventions may create some characteristics that differ from critically-ill medical patients. Therefore, this study was conducted to develop an AKI prediction score for critically-ill surgical patients to demonstrate the features of patients who have a greater chance of AKI following major non-cardiothoracic surgery and who are then admitted to the ICU.

## Methods

### Study design and the source of dataset

The prediction score was a secondary analysis from a prospective observational study, the Thai Surgical Intensive Care Unit (THAI-SICU) Study. It was conducted from 9-University base ICUs around Thailand between April 2010 to January 2013. Data was collected for 28 days following the ICU admission. A total of 4652 cases were collected with many outcomes of interest concerning complications following major non-cardiothoracic operations; for instance acute respiratory distress syndrome, delirium, and readmission, as which reported elsewhere [10–12].

One topic of interest from the THAI-SICU Study was AKI outcome. In summary, the incidence of AKI, using the Acute Kidney Injury Network (AKIN) classification, was at 16.9% [1] and incidence remained high at 19.3% when specified only in the elderly group, whose age was equal to or over 65 years old [12]. In the total cohort, renal replacement therapy (RRT) was commenced in about one-fifth (22.3%) of AKI patients. AKI is associated with bad outcomes including greater ICU mortality and 28-day mortality. The risk factors for developing AKI included a higher severity of illness as measured by APACHE-II scores, the presence of hypoalbuminemia, and organ dysfunction from the start of ICU admission.

### Inclusion/exclusion

Critically-ill surgical patients from the THAI-SICU Study with aged 18 and over who underwent major non-cardiothoracic surgery before admission to ICU were eligible for enrolment into the study. The exclusion criterion were patients admitted to the ICU for less than 24 h or who were admitted to the ICU due to medical rather than surgical reasons; for instance, congestive heart failure, volume overload, or exacerbation of airway diseases that had no association or correspondence with surgical interventions.

### Outcomes and definition of AKI

The primary outcome was the presence of AKI within first 7 days of ICU admission. AKI was defined according to the KDIGO criteria [13], which are an increase in serum creatinine (sCr) ≥ 0.3 mg/dL within 48 h or an increase of 1.5 times from baseline within a 7-day period.

The original dataset collected the incidence of AKI according to AKIN classification [1]. However, we extracted raw database that contained every single serum creatinine measurement during the ICU admission for reckoning AKI according to KDIGO criteria.

Reference sCr was selected according to the lowest sCr between the lowest value of sCr during ICU admission [14] or calculated back from MDRD equation by assuming patient’s baseline estimated glomerular filtration rate (eGFR) at 75 mL/min [15, 16]. In cases with a known history of chronic kidney disease, the best 3-month sCr preceding ICU admission was used as the reference value

Other secondary outcomes were also extracted: ICU mortality rate; day-28 mortality rate; ICU length of stay; and hospital length of stay.

### Predictors

Baseline characteristic data were utilized to deliver AKI prediction score including patient demographics (age, gender, body weight, and body mass index); pre-existing comorbidities (diabetes mellitus, hypertension, cardiovascular diseases, chronic pulmonary diseases, chronic kidney disease, malignancy, and others); severity of illness at ICU admission (measured by APACHE-II score, SOFA score, and SOFA non-renal score); sepsis at ICU admission; basic laboratory investigations at ICU admission (hemoglobin, serum albumin, blood sugar, PaO2/FiO2 ratio, chest imaging, electrocardiography, sCr, and reference sCr); and perioperative data before the ICU admission (including the American Society of Anesthesiologists [ASA] classification, emergency surgery, site of operation, perioperative time, blood loss, fluid balance, and urine output).

### Sample size

The effective sample size to enhance the statistical power in our study was calculated according to the most commonly mentioned “rule of thumb, 10 events needed per predictor” [17–19]. There were almost 32 possible predictors included in the model. That meant, the event of AKI should be about 320 (32*10) cases. The original dataset had an incidence of AKI at 16.9%. The suitable sample size enrolled to develop the scoring system should be at least 1893 (100/16.9*320) cases, in which cases from ours (4652 cases) were enough to build the model.

### Missing

Although we had tried hard to collect and clean data, missing values are inevitable. So, complete case analysis was used in our study.

### IRB committee and consent, TCTR

The Institutional Review Board’s approval for the study was obtained (Faculty of Medicine Vajira Hospital, Navamindradhiraj University, Bangkok, Thailand, COA 60/2561 and the Faculty of Medicine, Thammasat University, Pathumthani, Thailand, MTU-EC-ES-0-084/61), and internationally registered at http://www.clinicaltrials.in.th, TCTR20190408004. Informed consents were waived by reason of a secondary analysis of the dataset.

### Statistical analysis

Categorical data were expressed with frequencies (n) and percentages (%) and compared using Fisher’s exact test. Continuous data were presented in mean and standard deviation (SD) or median and interquartile range (IQR) and compared by Student’s *t*-test or Wilcoxon’s rank-sum test, as appropriate.

### Model development

To identify predictors that determined AKI, all predictors were first tested for multi-collinearity using variance inflation factor (VIF) > 10 criteria, and then entered into the model using multivariable logistic regression analysis. The possible significant variables were selected using criteria of *p*-value < 0.05 by backward elimination method. Categorization for continuous variables was done to facilitate odds ratio calculation.

### Score derivation and validation

The prediction score for each independent variable was created by calculating its multivariable logistic regression coefficients divided by the lowest value of the model and rounded to the nearest integer or 0.5. Each predictor score was summed up to a total AKI prediction score. The final score was tested for its discriminative ability using an area under the receiver operating characteristic curve (AuROC) or C-statistic [20]. Scoring calibration between predicted risk and observed risk were compared and presented graphically, and were tested by the Hosmer-Lemeshow Goodness-of-fit (HL-GOF) statistic. Internal validity was done by the bootstrapping method (1000 replications). Finally, the prediction scores were categorized into four levels of AKI probability: low, moderate, high, and very high risk. A positive likelihood ratio (LH+) of AKI and its 95%CI were reported for each level.

All analyses were performed using STATA statistical software version 13.0 (StataCorp LP, College Station, TX, USA) and *p*-values of less than 0.05 were considered statistically significant.

## Results

### Overview of AKI

A series of 4652 cases in THAI-SICU study were assessed for their eligibility. Patients aged below 18 (*n* = 28), medical reasons for surgical ICU admission (*n* = 998), and admitted to ICU less 24 h (*n* = 152) were excluded from the analysis. Study flow is provided in Additional file 1: Figure S1. Finally, 3474 cases were eligible for developing an AKI prediction score. Of these, 333 (9.6%) cases experienced AKI within 7 days of ICU admission.

In general, the AKI group were older, had more males, and a higher illness severity than the non-AKI group. No differences in patients’ pre-existing comorbidities were found between groups. More multiple abnormalities were identified on basic investigations in the AKI than in the non-AKI patients; including more anemia, less albuminemia, higher serum creatinine, lower PiO2/FiO2 ratio, abnormal chest imaging, and abnormal ECG. Regarding surgical intervention, AKI had higher class of ASA classification, more frequent emergency surgery, and had undergone more abdomino-colorectal surgery than non-AKI. A shorter duration of operative time, with a greater blood loss, and a lesser urine output were also found in AKI than in non-AKI. As for the outcomes, there was significantly greater risk of ICU and day-28 mortality in AKI than non-AKI, together with longer ICU length of stay and hospital length of stay in the AKI group (Table 1). Univariable logistic regression analysis relating each predictor to AKI is shown in Additional file 1: Table S1.

**Table 1:** Clinical characteristics comparing AKI vs non-AKI at ICU admission

Characteristics	AKI (*n* = 333)	Non-AKI (*n* = 3141)	*p*-value
Demographics			
Age – years	64.7 ± 17.1	61.9 ± 16.7	0.004
Female – n (%)	119 (35.7)	1380 (44.1)	0.004
Body weight – kg	59.8 ± 14.7	60.4 ± 17.2	0.558
Body mass index – kg/m^2^	23.0 ± 5.2	23.3 ± 6.0	0.413
Comorbidities			
Diabetes mellitus – n (%)	65 (19.5)	703 (22.4)	0.266
Hypertension – n (%)	162 (48.7)	1600 (50.9)	0.454
Cardiovascular diseases – n (%)	70 (21.0)	683 (21.7)	0.834
Respiratory diseases – n (%)	28 (8.4)	267 (8.5)	0.954
Chronic kidney disease – n (%)	35 (10.5)	287 (9.1)	0.426
Malignancies – n (%)	47 (14.1)	468 (14.9)	0.746
Others – n (%)	25 (7.5)	246 (7.8)	0.915
At ICU admission			
APACHE – II score	16 (11–21)	9 (6–13)	< 0.001
SOFA score	6 (3–9)	2 (0–4)	< 0.001
SOFA non-renal score	4 (2–8)	1 (0–3)	< 0.001
Sepsis at presentation – n (%)	115 (34.5)	214 (6.8)	< 0.001
Investigations			
Hemoglobin – gm/dL	10.0 ± 2.3	10.8 ± 2.0	< 0.001
Albumin - gm/dL	2.45 ± 0.75	2.83 ± 0.81	< 0.001
Blood sugar – mg/dL	164.6 ± 65.8	165.5 ± 55.0	0.784
PiO_2_/FiO_2_ ratio	276 ± 140	348 ± 124	< 0.001
Abnormal chest imaging – n (%)	84 (26.4)	417 (13.7)	< 0.001
Abnormal ECG – n (%)	122 (37.5)	707 (23.9)	< 0.001
Baseline creatinine – mg/dL*	1.02 (0.81–1.12)	0.81 (0.70–1.04)	< 0.001
Reference creatinine by – n (%)			< 0.001
History of renal insufficiency	70 (21.0)	510 (16.8)	
MDRD calculated back	203 (61.0)	872 (28.6)	
Lowest value of admission	60 (18.0)	1663 (54.6)	
Surgical interventions			
ASA classification -n (%)			< 0.001
I	7 (2.2)	207 (6.8)	
II-III	219 (68.4)	2521 (82.7)	
IV-V	94 (29.4)	319 (10.5)	
Emergency surgery – n (%)	222 (68.3)	833 (28.1)	< 0.001
Site of surgery – n (%)			
Neuro, head, and neck	11 (3.3)	392 (12.5)	< 0.001
Abdomen & colorectal	232 (69.7)	1846 (58.8)	< 0.001
Orthopedics	43 (12.9)	458 (14.6)	0.460
Others	63 (18.9)	514 (16.4)	0.245
Operative time – min^a^	155 (90–270)	240 (150–345)	< 0.001
Perioperative blood loss – mL^a^	500 (150–2000)	450 (200–1050)	0.257
Perioperative fluid balance – mL^a^	1400 (665–2720)	1707 (800–2832)	0.067
Perioperative urine output – mL^a^	180 (40–450)	343 (130–695)	< 0.001
Outcomes			
ICU mortality – n (%)	85 (25.5)	90 (2.9)	< 0.001
Death at day-28 – n (%)	109 (32.7)	174 (5.5)	< 0.001
ICU length of stay - days^a^	6 (3–13)	1 (1,3)	< 0.001
Hospital length of stay – days*	18 (10–28)	14 (9–24)	0.003

Continuous data were reported as mean ± SD. Otherwise, data with ^a^ were reported as median and IQR 1–3

*ICU* Intensive care unit, *APACHE-II score* Acute Physiology and Chronic Health Evaluation II score, *SOFA score* Sequential Organ Failure Assessment score, *ECG* Electrocardiogram, *MDRD* The Modification of Diet in Renal Disease

### Predictors that determined AKI within 7 days of ICU admission

**Fig. 1:**
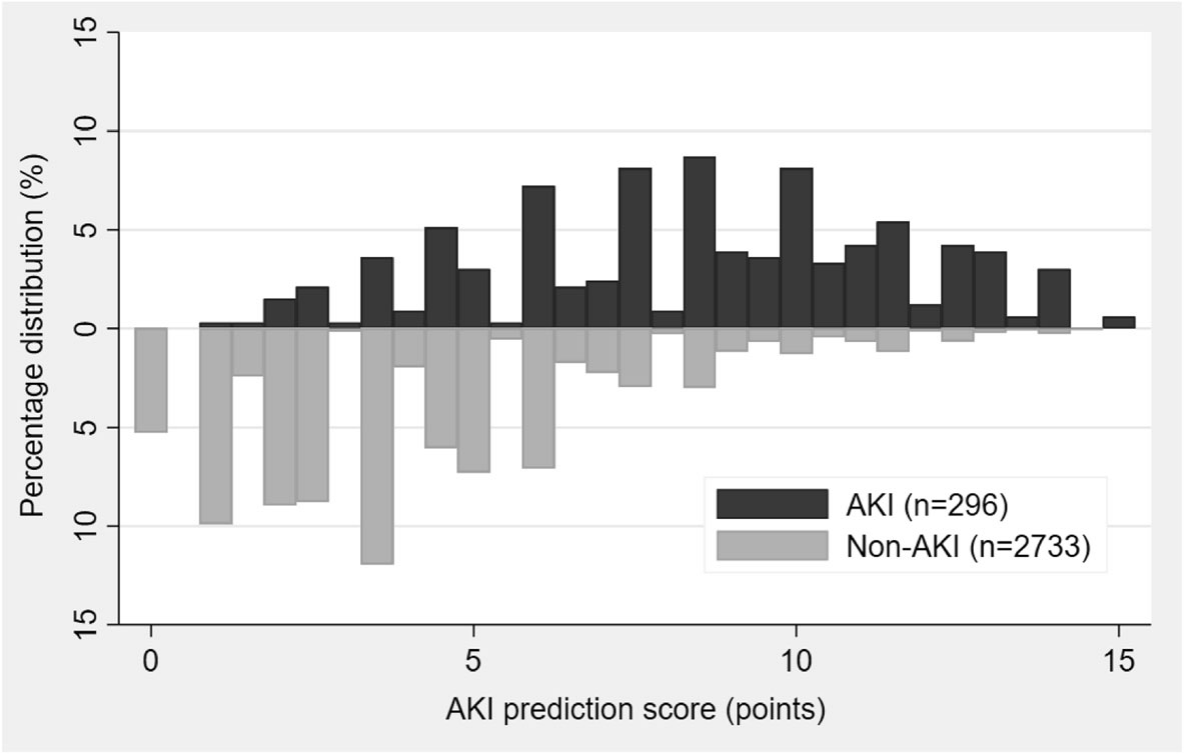
Percentage distribution of each AKI prediction score categorized by AKI and non-AKI

**Fig. 2:**
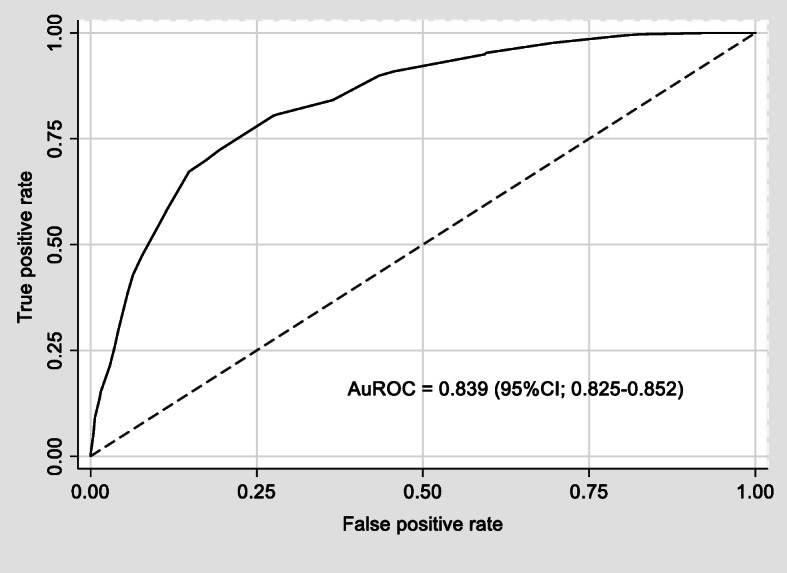
The discriminative ability of acute kidney injury prediction score in critically-ill surgical patients reported by Area under the Receiver Operating Characteristic Curve (AuROC)

Table 2 shows the best AKI predictors using multivariable logistic regression analysis. The final selected predictors included age of patient, SOFA non-renal score, sepsis, emergency surgery, perioperative blood loss, and perioperative urine output. After arranging into a scoring system, the AKI prediction score was ranged between 0 to 16.5. Figure 1 illustrates the number of cases distributed according to each score level comparing AKI and non-AKI. The AKI prediction score had a good discriminative ability with AuROC = 0.839; 95%CI, 0.825–0.852 (Fig. 2), and fitted to the original dataset by HL-GOF, *p* value = 0.302. The sensitivity, specificity, positive predictive value (PPV), and negative predictive value (NPV) of the score was 72.3, 80.6, 28.8, and 96.4%, respectively. The model remained a good discriminative ability, AuROC = 0.821; 95%CI, 0.797–0.845, after internal validating by bootstrapping method (1000 replications).

**Table 2:** Best multivariable risk predictors that determined acute kidney injury

Risk factor	OR	95%CI	*p*-value	Coefficient	Score
Age < 65	Ref				0
Age over 65	1.611	1.219–2.128	0.001	0.477	1
SOFA non-renal at ICU admission					
0–1	Ref				0
2–5	2.690	1.871–3.868	< 0.001	0.990	2.5
≥ 6	7.393	4.899–11.157	< 0.001	2.001	5.0
No sepsis at ICU admission	Ref				0
Sepsis at ICU admission	3.397	2.426–4.757	< 0.001	1.223	3.0
Elective surgery	Ref				0
Emergency surgery	2.457	1.813–3.332	< 0.001	0.899	2.5
Perioperative blood loss					
< 1000 mL	Ref				0
≥ 1000–2500 mL	1.708	1.175–2.482	0.005	0.535	1.5
> 2500 mL	2.839	1.909–4.220	< 0.001	1.043	2.5
Perioperative urine output					
≥ 500 mL	Ref				0
100–499 mL	1.149	1.034–2.086	0.032	0.384	1
< 100 mL	2.374	1.628–3.461	< 0.001	0.865	2.5

**Fig. 3:**
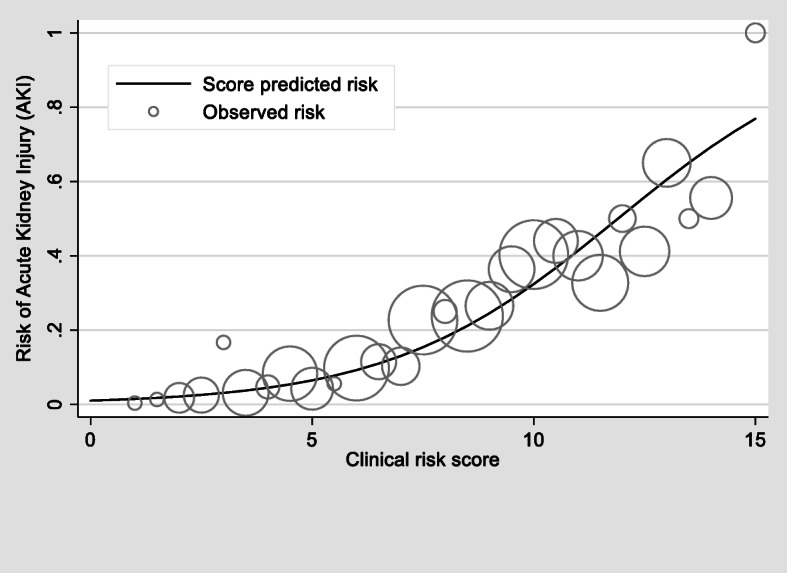
Observed risk (circle) vs predicted risk (solid line) of AKI, the size of circle represents frequency of patients in each score level

The higher the score the greater the risk of AKI, and the predicted risk of the score was closely correlated with the reality (observed risk), as shown graphically (Fig. 3). We then classified the score into fourth probability risk of AKI; as low, moderate, high, and very high risk. The LH+ of AKI was at 0.117, 0.927, 5.190, and 9.982, respectively (Table 3).

**Table 3:** Distribution of AKI vs non-AKI categorized by probabilities

Probability categories	Score	AKI (*n* = 296)	Non-AKI (*n* = 2733)	LH+	95%CI	*p*-value
Low	0–2.5	14 (4.7)	1107 (40.5)	0.117	0.063–0.200	< 0.001
Moderate	3.0–8.5	142 (48.0)	1415 (51.8)	0.927	0.745–1.148	0.477
High	9.0–11.5	95 (32.1)	169 (6.2)	5.190	3.881–6.910	< 0.001
Very High	12.0–16.5	45 (15.2)	42 (1.5)	9.892	6.230–15.695	< 0.001
Mean ± SD		8.5 ± 3.2	4.1 ± 2.9			< 0.001

*LH+* Positive likelihood ratio

## Discussion

According to a nation-wide, multicenter surgical ICUs dataset – the THAI-SICU Study, in a week after a major non-cardiothoracic operation, patients who stayed in surgical ICUs, nearly 10% of them suffered AKI, with an almost 10-times greater risk of ICU mortality than non-AKI patients. Moreover, AKI in critically-ill surgical patients could be simply predicted by the means of just six simple pre-ICU demographics, combining both patient baseline characteristics and perioperative data. The predictors that determine AKI are patient age, SOFA non-renal score, sepsis at ICU admission, emergency surgery, peri-operative blood loss, and peri-operative urine output. The last three predictors collected from the perioperative period made the score unique for critically-ill surgical patients, and have rarely been reported before [21]. In a total score of 16.5, increasing the score increases the probability of AKI.

Our AKI prediction score has a good discriminative ability (c-statistic of 0.839; 95%CI, 0.825–0.852 and 0.821; 95%CI, 0.797–0.845, after internal validating by bootstrapping). Previous studies about AKI prediction score, which studied in different populations and timing of prediction, have usually reported good diagnostic function. Most of them range above 0.80. For instance, the study form patients who had undergone liver resection, in their development cohort, AKI prediction score’s C-statistic was at 0.81 (95%CI, 0.76–0.86) [22]. Another study from Kheterpal et al., who built a scoring system for predicting AKI following major general surgery (not specify only critically-ill surgical patients), reported a good diagnostic model with of 0.80 (95%CI, 0.79–0.81) [8]. Another study by Malhotra et al. stated just a moderate to good function of their AKI prediction model, at 0.79 (95%CI, 0.70–0.89) [9]. However, their study populations were mixed both medical and surgical critically ill patients.

The AKI prediction scores in patients who underwent major operation have been reported from other settings. For instance, the AKI prediction score reported by Bell and colleagues [23]. They addressed an importance of AKI prediction score in orthopedic surgical patients and its impact on short and long-term survival outcomes. The AKI predictive ability of their score was (AuROC) 0.74 (95%CI, 0.73–0.75) in the derivative cohort and 0.73 (no 95%CI reported) when internally validated. However, all of the predictors were only derived from preoperative data, without any aggregated data regarding peri-operation and severity of illness after an operation. The other two AKI prediction scores were reported from general major non cardiothoracic surgery, not specified only critically ill patient, by Park et al [24] and Lei et al. [25] The study by Park and colleagues [24] reported quite good AKI prediction ability, an AuROC of 0.80 (95%CI, 0.79–0.81) in the derivation cohort, but decreased slightly to 0.72 (95%CI, 0.71–0.73) when externally validated. However, this study used only preoperative data for developing the AKI prediction score. Another study by Lei and colleagues [25], they demonstrated an AuROC of 0.712 (95%CI, 0.694–0.731), when the score was derived from the pre-operative data. When added peri-operative and post-operative data to pre-operative data, a significant increase in model performance was found (*p* < 0.001). The AuROC increased to be 0.804 (95%CI, 0.788–0.899) and 0.871 (95%CI, 0.802–0.832), respectively. The results from this study confirmed our concern regarding the importance of peri-operative and post-operative data should be co-operated into the AKI prediction score.

The diagnostic indices, comprising sensitivity, specificity, PPV, and NPV, in our prediction score were 72.3, 80.6, 28.8, and 96.4%, respectively. A high percentage of NPV made our score beneficial for including most of the patients who are at risk of AKI. Thus, fewer cases will be missed by our prediction score. Moreover, the diagnostic properties of our study were quite similar to studies from Malhotra et al. (74, 72, 23, and 96%, respectively) [9] and Rueggeberg et al. (78, 92, 62, and 96%, respectively) [26]. However, it might be very difficult to interpret differences between prediction models in the details, because of diverse definitions of some variables and study populations.

The predictors that determine AKI in our study were comparable to previous studies. For instance, patient age [8, 24, 27], sepsis at ICU admission [9], SOFA non-renal score [1, 28], emergency surgery [8, 24], and perioperative blood lost [29].

Increased patient age increased the risk of AKI. However, with some differences in the cut-off value, our used 65, most frequently and acceptably used, whereas, in mixed critically-ill patients, age of ≥56 years was used [8]. Somehow, in another study created the scores corresponding the increase in ranges of age [24].

The report from mixed critically-ill patients by Malhotra et al. showed that severe infection and sepsis were associated with AKI [9]. However, in major non-cardiac surgery studies, sepsis was lacking as one of AKI predictors [8, 22–24, 27].

The spectrum of illness severity, as measured by SOFA non-renal score, was included as one of our predictors. To the best of our knowledge, no preceding studies contained severity of illness in their AKI prediction scores. The use of SOFA non- renal score, after categorization into 3 levels (0–1, 2–5, and ≥ 6), represented the risk of AKI, sequentially. We excluded SOFA renal score domain to eliminate the effect of individual baseline renal function on ICU admission from total SOFA score, as per some recommendations from previous reports [1, 28].

Regarding surgical information, emergency surgery was undoubtfully found as one of the AKI prediction score, similar to the previous studies [8, 24]. Other perioperative risk factors including peri-operative blood loss and peri-operative urine output, recently, there has been no definitive consensus on how much blood loss is correlated to the risk of AKI. However, a study by Kim et al [30], showed that every 1 l of perioperative blood loss in liver transplant recipients increased the risk of continuous renal replacement therapy significantly. As for urine output during operation, there were some differences in our data compared to other study. Slankamenac K et al. found oliguria raised the possibility of AKI [22], they defined oliguria as urinary output < 400 ml/24 h. Though, in our study, we arranged perioperative differently.

To the best of our knowledge, our study represents one of the largest series of AKI in critically-ill surgical patients who underwent major non-cardiothoracic surgery. The availability of intensive monitoring for every case in the ICU might be difficult in some centers, particularly in resource-limited countries, such as Thailand. This AKI prediction score can be utilized in daily clinical practice for early AKI detection. Selected cases that are at high risk of AKI will benefit from more frequent serum creatinine blood sampling, hourly urine output measurement, aggressive fluid resuscitation, optimization of fluid balance management, and avoidance of unnecessary nephrotoxic agents to mitigate the occurrence of AKI and to augment renal function recovery.

### Limitations

There were some limitations in our study. First, AKI was diagnosed based on serum creatinine only. This could underestimate the overall incidence of AKI. Urine output is another criterion for an AKI diagnosis, but it was not used because of the lack of information on this variable available from the THAI-SICU Study. Second, the AKI prediction score was only able to determine AKI in a period of 1 week following ICU admission. It was thought to be based upon the fact that how long does perioperative AKI has no clear definition [27], and how long this usually acute disease lasted for after the operation is unknown. Moreover, during ICU admissions of more than a week, AKI may occur due to other factors; for instance, nosocomial infection, surgical site infection, or an exposure to nephrotoxic agents. Third, perioperative urine output was categorized into 3 orders. The urine output should be adjusted by body weight and perioperative period. We attempted to use urine output/kg/hour as the predictor, but the results after testing by statistical analysis showed that an ordination of raw urine output was more suitable and had more discriminative ability for AKI prediction than urine output/kg/hour. Finally, the intraoperative hypotension was not accounted for in out AKI predictors due to a lack of this information in our dataset. As previous studies had been shown that intraoperative hypotension has significantly impacted on the occurrence of AKI post-operatively [31, 32].

### Further study

We hope to apply the AKI prediction score into our clinical practice. It would be of great value in validating the scoring system in critically-ill surgical patients from other centers. Moreover, some prediction scores for predicting other kidney issues in the ICU might be topics of interest, such as a score for predicting patients who will benefit from commencing early replacement therapy, or a score for forecasting patients who will experience renal function recovery.

## Conclusions

The diagnostic function of the AKI prediction score for predicting AKI in critically-ill patients who underwent major non-cardiothoracic surgery was good.

The AKI prediction score could offer clinicians early identification of critically-ill surgical patients who are at risk of AKI from the start of ICU admission. Decision making for more aggressive treatment to prevent or to treat AKI may be guided by the score from the operative period or upon admission to ICU.

## Supplementary information

**Additional file 1 Figure S1.** Study flow. **Table S1.** Univariable logistic regression analysis on variables to predict the occurrence of AKI.

## Data Availability

The datasets used and/or analysed during the current study are available from the corresponding author on reasonable request.

## Abbreviations

AKI: Acute kidney injury
AKIN classification: The Acute Kidney Injury Network classification
APACHE-II score: Acute Physiology and Chronic Health Evaluation II score
ASA classification: American Society of Anesthesiologists classification
ECG: Electrocardiogram
eGFR: Estimated glomerular filtration rate
HL-GOF: Hosmer-Lemeshow Goodness-of-fit statistic
ICU: Intensive care unit
KDIGO: The Kidney Disease Improving Global Outcomes-2012
LH +: Likelihood ratios of positive
MDRD: The Modification of Diet in Renal Disease
NPV: Negative predictive value
NGAL: Neutrophil gelatinase-associated lipocalin
PPV: Positive predictive value (PPV)
AuROC: The area under Receiver Operating Curve
RRT: Renal replacement therapy
sCr: Serum creatinine
SOFA score: Sequential Organ Failure Assessment score
THAI-SICU Study: Thai Surgical Intensive Care Unit Study
