# Supplementary material for: Acute kidney injury risk prediction score for critically-ill surgical patients
Source: BMC Anesthesiol. 2020 Jun 3;20:140. doi: 10.1186/s12871-020-01046-2 (PMC7271390; doi:10.1186/s12871-020-01046-2)
Supplement: Supplementary file 1 — Additional file 1 Figure S1. Study flow. Table S1. Univariable logistic regression analysis on variables to predict the occurrence of AKI. [file 12871_2020_1046_MOESM1_ESM.docx]

# Additional File 1

## Figure S1. Study flow

## Table S1. Univariable logistic regression analysis on variables to predict the occurrence of AKI.

Excluded (n= 1178)

- Age < 18 (n=28)

- Medical reasons for admission (n=998)

- ICU admission < 24 hr (n=152)

Patient who underwent major non-cardiothoracic surgery before the ICU admission (n=3474)

Clinical characteristics (n=3474)

- Age, gender, BW, BMI

- Co-morbidities

- APACHE-II, SOFA, and SOFA non-renal

- Sepsis at ICU admission

- Laboratory investigations

- Surgical interventions

ASA classification

Emergency surgery

Site of surgery

Operative time

Perioperative blood loss

Perioperative urine output

Perioperative fluid balance

Acute kidney injury within 7 days (n=333)

No Acute kidney injury within 7 days (n=3141)

Critically ill surgical patients from THAI-SICU Study dataset (n=4652)

## Figure S1 Study flow

## Table S1 Univariable logistic regression analysis on variables to predict the occurrence of AKI.

| Characteristics | AKI  (n=333) |  | Non-AKI  (n=3141) |  | OR | 95%CI | p-value |  |
| --- | --- | --- | --- | --- | --- | --- | --- | --- |
| Demographics |  |  |  |  |  |  |  |  |
| Age – years | 64.7±17.1 | | 61.9±16.7 | | 1.010 | 1.003-1.018 | 0.004 |  |
| Female – n (%) | 119 (35.7) | | 1380 (44.1) | | 0.706 | 0.558-0.893 | 0.004 |  |
| Body Weight – kg | 59.8 (14.7) | | 60.4 (17.2) | | 0.998 | 0.991-1.005 | 0.558 |  |
| Body Mass Index – kg/m^2^ | 23.0±5.2 | | 23.3±6.0 | | 0.992 | 0.972-1.012 | 0.413 |  |
| Comorbidities |  |  |  |  |  |  |  |  |
| Diabetes mellitus – n (%) | 65 (19.5) | | 703 (22.4) | | 0.841 | 0.634-1.117 | 0.266 |  |
| Hypertension – n (%) | 162 (48.7) | | 1600 (50.9) | | 0.912 | 0.728-1.144 | 0.454 |  |
| Cardiovascular diseases – n (%) | 70 (21.0) | | 683 (21.7) | | 0.958 | 0.726-1. 263 | 0.834 |  |
| Respiratory diseases – n (%) | 28 (8.4) | | 267 (8.5) | | 0.988 | 0.484-0.515 | 0.954 |  |
| Chronic kidney disease – n (%) | 35 (10.5) | | 287 (9.1) | | 1.168 | 0.806-1.691 | 0.412 |  |
| Malignancies – n (%) | 47 (14.1) | | 468 (14.9) | | 0.938 | 0.679-1.297 | 0.701 |  |
| Others – n (%) | 25 (7.5) | | 246 (7.8) | | 0.955 | 0.623-1.465 | 0.834 |  |
| At ICU admission | |  |  |  |  |  |  |  |
| APACHE – II score | 17.0±7.6 | | 9.9±5.6 | | 1.159 | 1.139-1.179 | <0.001 |  |
| (median, IQR 1,3) | 16 (11-21) | | 9 (6-13) | |  |  |  |  |
| SOFA score | 6.2±3.8 | | 2.4±2.6 | | 1.385 | 1.338-1.433 | <0.001 |  |
| (median, IQR 1,3) | 6 (3-9) | | 2 (0-4) | |  |  |  |  |
| SOFA non-renal score | 5.0±3.6 | | 1.9±2.3 | | 1.381 | 1.332-1.433 | <0.001 |  |
| (median, IQR 1,3) | 4 (2-8) | | 1 (0-3) | |  |  |  |  |
| Sepsis at ICU admission– n (%) | 115 (34.5) | | 214 (6.8) | | 7.215 | 5.535-9.406 | <0.001 |  |
| Investigations |  |  |  |  |  |  |  |  |
| Hemoglobin – gm/dL | 10.0±2.3 | | 10.8±2.0 | | 0.838 | 0.794-0.885 | <0.001 |  |
| Albumin - gm/dL | 2.45±0.75 | | 2.83±0.81 | | 0.549 | 0.467-0.645 | <0.001 |  |
| Blood sugar – mg/dL | 164.6±65.8 | | 165.5±55.0 | | 0.999 | 0.997-1.002 | 0.784 |  |
| PiO_2_/FiO_2_ ratio | 276±140 | | 348±124 | | 0.998 | 0.997-0.998 | <0.001 |  |
| Abnormal chest imaging – n (%) | 84 (26.4) | | 417 (13.7) | | 2.261 | 1.727-2.962 | <0.001 |  |
| Abnormal ECG – n (%) | 122 (37.5) | | 707 (23.9) | | 1.910 | 1.503-2.428 | <0.001 |  |
| Baseline creatinine – mg/dL | 1.17±0.80 | | 1.21±1.48 | | 1.019 | 0.936-1.110 | 0.662 |  |
| (median, IQR 1,3) | 1.02 (0.81-1.12) | | 0.81 (0.70-1.04) | |  |  | <0.001 |  |
| Reference creatinine by – n (%) |  |  |  |  |  |  |  |  |
| History of renal insufficiency | 70 (21.0) | | 510 (16.8) | |  |  |  |  |
| MDRD calculated back | 203 (61.0) | | 872 (28.6) | |  |  |  |  |
| Lowest value of admission | 60 (18.0) | | 1663 (54.6) | |  |  |  |  |
| Surgical interventions |  |  |  |  |  |  |  |  |
| ASA classification -n (%) |  |  |  |  |  |  |  |  |
| I | 7 (2.2) | | 207 (6.8) | | Ref | Ref | Ref |  |
| II-III | 219 (68.4) | | 2521 (82.7) | | 2.569 | 1.194-5.525 | 0.016 |  |
| IV-V | 94 (29.4) | | 319 (10.5) | | 8.714 | 3.964-19.153 | <0.001 |  |
| Emergency surgery – n (%) | 222 (68.3) | | 833 (28.1) | | 5.516 | 4.309-7.060 | <0.001 |  |
| Site of surgery – n (%) |  |  |  |  |  |  |  |  |
| Neuro, head, and neck | 11 (3.3) | | 392 (12.5) | | 0.240 | 0.130-0.441 | <0.001 |  |
| Abdomen & colorectal | 232 (69.7) | | 1846 (58.8) | | 1.611 | 1.262-2.057 | <0.001 |  |
| Orthopedics | 43 (12.9) | | 458 (14.6) | | 0.869 | 0.621-1.215 | 0.460 |  |
| Others | 63 (18.9) | | 514 (16.4) | | 1.193 | 0.892-1.594 | 0.245 |  |
| Operative time (min) | 209±164 | | 268±164 | | 0.997 | 0.996-0.998 | <0.001 |  |
| (median, IQR 1,3) | 155 (90-270) | | 240 (150-345) | |  |  | <0.001 |  |
| Perioperative blood loss (mL) | 1605±2837 | | 957±1596 | | 1.000 | 1.000-1.000 | <0.001 |  |
| (median, IQR 1,3) | 500 (150-2000) | | 450 (200-1050) | |  |  | 0.257 |  |
| Perioperative fluid balance (mL) | 1974±2176 | | 2028±1796 | | 1.000 | 0.999-1.000 | 0.606 |  |
| (median, IQR 1,3) | 1400 (665-2720) | | 1707 (800-2832) | |  |  | 0.067 |  |
| Perioperative urine output (mL) | 304±367 | | 504±586 | | 0.999 | 0.998-0.999 | <0.001 |  |
| (median, IQR 1,3) | 180 (40-450) | | 343 (130-695) | |  |  | <0.001 |  |
